# Engineering IgG-Like Bispecific Antibodies—An Overview

**DOI:** 10.3390/antib7030028

**Published:** 2018-08-01

**Authors:** Simon Krah, Harald Kolmar, Stefan Becker, Stefan Zielonka

**Affiliations:** 1 Protein Engineering and Antibody Technologies, Merck KGaA, Frankfurter Strasse 250, D-64293 Darmstadt, Germany; stefan.c.becker@merckgroup.com; 2 Institute for Organic Chemistry and Biochemistry, Technische Universität Darmstadt, Alarich-Weiss-Strasse 4, D-64287 Darmstadt, Germany; kolmar@biochemie-TUD.de

**Keywords:** bispecific antibodies, cognate light chain pairing, common heavy chain, common light chain, heavy chain heterodimerization, knobs into holes, SEED

## Abstract

Monoclonal antibody therapeutics have proven to be successful treatment options for patients in various indications. Particularly in oncology, therapeutic concepts involving antibodies often rely on the so-called effector functions, such as antibody dependent cellular cytotoxicity (ADCC) and complement dependent cytotoxicity (CDC), which are programed in the antibody Fc region. However, Fc-mediated effector mechanisms often seem to be insufficient in properly activating the immune system to act against tumor cells. Furthermore, long term treatments can lead to resistance against the applied drug, which is monospecific by nature. There is promise in using specific antibodies to overcome such issues due to their capability of recruiting and activating T-cells directly at the tumor site, for instance. During the last decade, two of these entities, which are referred to as Blinatumomab and Catumaxomab, have been approved to treat patients with acute lymphoblastic leukemia and malignant ascites. In addition, Emicizumab, which is a bispecific antibody targeting clotting factors IXa and X, was recently granted market approval by the FDA in 2017 for the treatment of hemophilia A. However, the generation of these next generation therapeutics is challenging and requires tremendous engineering efforts as two distinct paratopes need to be combined from two different heavy and light chains. This mini review summarizes technologies, which enable the generation of antibodies with dual specificities.

## 1. Introduction

The therapeutic potential of monoclonal antibodies (mAbs) is enormous but may be limited due to the simple fact that they are only able to bind one distinct target, while complex diseases in general originate from multiple factors and mediators [1]. In contrast, bispecific antibodies (bsAbs) consist of two physically connected antigen binding moieties (i.e., paratopes), which can simultaneously interact with different epitopes on the same or on different antigens [2,3,4,5]. The most established mode of action of these entities is the redirection of T-cells to tumors [6]. While one paratope of the so-called “T-cell engagers” binds a tumor antigen, the other specificity is directed against a part of the T-cell receptor termed CD3. By clustering of CD3 at the tumor site in a target specific manner, the T-cell becomes activated and consequently, the formation of a cytolytic synapse leads to the killing of the tumor cell [7]. The clinical effectiveness of this concept led to the market approval of the antibodies Catumaxomab in 2009 (Fresenius SE & Co. KGaA, withdrawal in 2017 for commercial reasons) and Blinatumomab (Amgen Inc.) in 2014. In addition to T-cell engagement, the common light chain-based bsAb Emicizumab (F. Hoffmann-La Roche AG) was approved for the treatment of hemophilia A by authorities in the US in 2017. This antibody is a mimetic of blood clotting factor VIII, which is not present in hemophilia patients due to a genetic defect [8,9]. By crosslinking factors IX and X, the bsAb restores this part of the coagulation cascade. In addition to these marketed therapeutics, about 30 drugs based on bispecific binding are in clinical development and approximately 60 of these entities are scrutinized in preclinical evaluation [10]. Among these, several therapeutic concepts and modes of action are investigated, such as the dual blocking of signaling pathways. Roche’s Duligotzumab is a phage-derived human antibody, which is engineered to bind two antigens with one heavy chain and light chain combination [6,11,12]. This so-called “Dual Action Fab” (DAF) is a two-in-one antibody, which can bind to EGFR and Her3. Deregulated signaling of both receptor tyrosine kinases (RTKs) is involved in the pathogenesis of cancers, such as colorectal and head and neck cancer [13,14]. Resistance mechanisms observed with the standard of care Cetuximab might be circumvented by the application of Duligotzumab [6]. Another molecule that follows this targeting strategy was developed by Merrimack Pharmaceuticals. MM-111 consists of Her2 and Her3 targeting single chain Fv fragments (scFv), where the variable domains of the heavy and light chain are connected by a linker and fused to modified human serum albumin (HSA) for extension of half-life [4,15]. While Her3 signaling is an important resistance mechanism to Her2 inhibitors, dual targeting of both RTKs can enhance an effective tumor response. Moreover, bsAbs are validated as tumor angiogenesis inhibitors. The CrossMab RG7221 targets the two key angiogenic proteins vascular endothelial growth factor A (VEGF-A) and angiopoetin-2 (Ang-2). The neutralization of two angiogenic factors is expected to lead to superior clinical outcomes compared to monospecific targeting [16]. In the context of immunology, several bsAbs capturing two different cytokines were developed. One example is M1095/ALX-07613 (Merck KGaA; Ablynx), which is a trivalent bispecific single-domain antibody fragment from camelids (VHH, nanobody) neutralizing both Il17-A and IL17-F for the treatment of inflammatory diseases. Extension of half-life is mediated by an additional VHH domain binding to human serum albumin [17,18].

Apart from clinical evaluations, novel targeting strategies have emerged using bsAbs. In a proof of concept study, which was recently published by our group, we demonstrated that affinity-optimized paratopes can be used to enhance the selectivity and efficacy of bsAbs and bsAb drug conjugates. In detail, lower affinity variants of humanized Cetuximab in an EGFR and cMet targeting bsAb showed reduced binding to keratinocytes (representing healthy tissue) while maintaining effective tumor cell killing [19].

All in all, bsAbs are promising molecules, which might overcome some of the therapeutic limitations experienced with conventional mAbs. However, the generation of these molecules is challenging and requires extensive protein-engineering and development of the manufacturing process depending on the chosen antibody format. A plethora of such formats is described in literature, which are elegantly reviewed elsewhere [6,20]. A general distinction can be made between IgG- and non-IgG-like bispecifics. The latter do not comprise a Fc-portion and are mainly made up from a single polypeptide chain, which eases their upstream and downstream processing. Consequently, these molecules are not able to facilitate Fc-mediated effector functions and show a faster clearance from the body due to a lack of a FcRn binding site. While IgG-like bispecifics are equipped with these Fc-based antibody features, a correct pairing of different polypeptide chains needs to be accomplished during manufacturing [3]. This mini review gives a general overview of methodologies that can be used to engineer such IgG-like bispecific entities.

## 2. The Chain Association Issue

**Figure 1:**
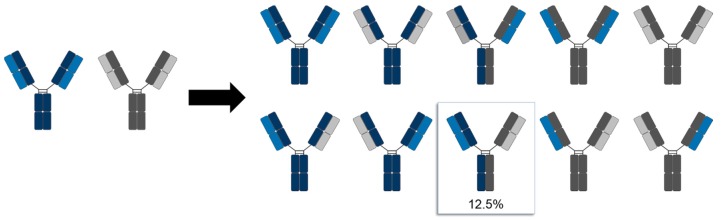
Schematic illustration of the bsAb chain association issue. Upon co-transfection of a host-cell with plasmids encoding for two different heavy and two different light chains, ten unique chain pairings can occur. The desired bsAb will appear with a statistical probability of 12.5%.

Conventional antibodies are formed from two identical heavy and two identical light chains. The co-transfection of a host cell with two different plasmids (one encoding for the heavy and one for the light chain) will always result in antibodies with correctly assembled heavy and light chains. With regards to IgG-like bispecifics, however, individual plasmids encoding for two different heavy and two different light chains need to be utilized for transfection. This leads to a significant fraction of mispaired antibodies after production, because each heavy chain can either homo- or heterodimerize and can in turn associate with both light chains. In total, ten unique chain combinations can associate, from which only one will be the bsAb with properly assembled heavy and light chains (Figure 1) [2,3]. Apart from the stepwise purification of the desired bispecific molecule via affinity columns with immobilized antigens, it is difficult to get rid of undesired side-products during downstream processing.

Moreover, the analytical characterization of antibody mixtures remains challenging. The standard process includes liquid chromatography (LC) coupled with mass spectrometry (MS). Recently, Genentech published strategies to efficiently characterize mispaired antibody fractions using reversed phase high-performance LC and Orbitrap-based high-resolution LC–MS. This platform allows the robust and sensitive analyses of hundreds of different samples [21,22].

## 3. Methods for Heavy Chain Heterodimerization

**Figure 2:**
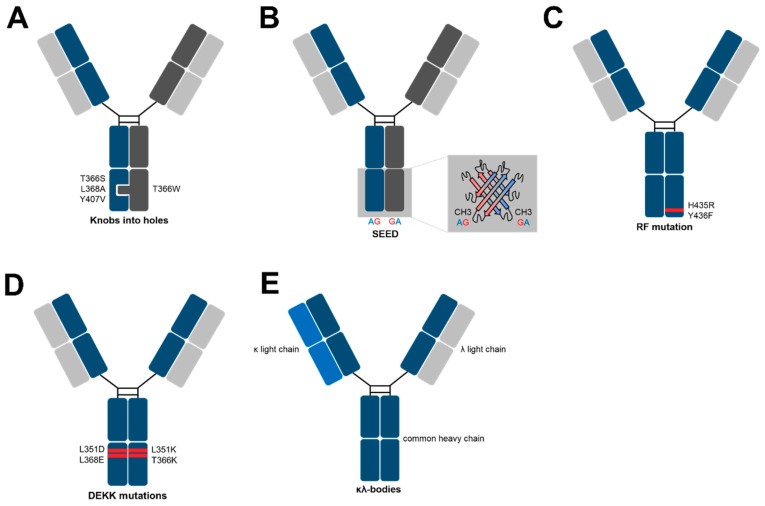
Methods for specific heavy chain association in IgG-like bsAbs.

Historically, the first bsAbs were generated by either chemical coupling of Fab-fragments or Hybrid–Hybridomas [23,24,25,26]. While coupled antibody fragments lacked a Fc portion (i.e., absence of recycling and effector mechanisms) and were difficult to produce, the latter did not yield bsAbs in sufficient quantities due to the mispairing of different heavy and light chains. In 1996, Carter and colleagues presented the so-called “knobs-into-holes” (KIH) technology [27]. Originally, KIH were proposed as a model for the packing of amino acid side chains in α-helices by Crick in 1953 [28]. In the context of bsAbs, it was used as a design strategy to promote the formation of heterodimers when expressing two different heavy chains (Figure 2A). In detail, mutations were incorporated into interface residues within the CH3 domain. In the knob CH3, small amino acid side chains were substituted by larger ones (tyrosine and tryptophan, e.g., mutation T366Y), while the counterpart was generated in another CH3 domain by mutating bulky to small amino acids, such as alanine and threonine (e.g., mutation Y407T). This rational design strategy was afterwards refined using phage display screening, in which a CH3 knob mutant (T366W) was paired with a library of variants that contained random mutations at the interface positions on the opposite chain. The fusion of the library of CH3 variants to M13 pIII in combination with the flag-tagged knob variant allowed the specific isolation of heterodimeric heavy chains, which resulted in more stable mutations compared to the previously designed KIHs (T366S; L368A; Y407V) [29]. Moreover, the incorporation of cysteine residues, which gives rise to inter CH3 disulfide bonds, further enhanced the heterodimerization in KIH heavy chains [30]. Taking together, along with a common light chain-based phage display screening, Genentech were the first to present a comprehensive platform for the generation of IgG-like bispecifics. Upon co-expression in mammalian cells, bsAbs were predominantly produced, although the resulting molecules are formed from separate polypeptide chains. During the last two decades, several other technologies for correct heavy chain pairing in bsAbs have been published and a few examples will be described more in detail in the following section.

An alternative approach for the specific association of different heavy chains is the use of the so-called SEEDbodies, in which SEED stands for “Strand Exchanged Engineered Domains” (Figure 2B) [31]. Herein, Fc heterodimerization is driven by interdigitating β-strand segments in the CH3 of a human IgG and IgA, resulting in the two hybrid chains termed SEED-GA and SEED-AG. In general, A-G or G-A crossover points were chosen by a structural alignment of the IgA and IgG domains along their 2-fold axis of symmetry. Shared “crossover points” were employed to change the order and composition of alternating β-strands.

One issue faced by protein engineers during the development of SEEDbodies was associated with the fact that FcRn and Protein-A binding sites are located within the junction of CH2 and CH3 domains in IgG while they are not present in IgA molecules. FcRn is required for establishing an extended half-life, while Protein-A is commonly used for antibody purification. Molecular modeling led to the suggestion that the SEED-AG would not associate to those proteins anymore. Therefore, solvent-exposed residues in that region were returned to IgG. Mouse pharmacokinetic studies and Protein-A retention times revealed a similar profile of refined SEEDbodies compared to conventional SEEDbodies [31]. The therapeutic assessment of this technique was demonstrated one year later by a head-to-head comparison of mono- or bivalent C225 (anti-EGFR) based SEEDs and the clinically validated C225 antibody in terms of in vivo half-life, stability and the potential to mediate effector functions (ADCC/CDC) with similar outcomes in all disciplines [32].

Another very elegant approach to get rid of any homodimeric side products during antibody-purification is the incorporation of only two mutations into the CH3 domain of one heavy chain. This method stands out with its low amount of protein engineering needed to specifically isolate heavy chain heterodimers. Moreover, the heavy chains remain nearly unchanged, which is an attribute that might be beneficial with regards to immunogenicity. In more detail, it is well known that IgG3 is not able to bind Protein-A [33]. The transfer of the two IgG3 CH3 amino acids, which are namely arginine and phenylalanine, to a IgG1 CH3 (positions: H435R and Y436F) prevents this chimera from binding to a Protein-A resin. Taking this into consideration, Regeneron recently published a focused strategy to generate bsAbs by incorporating the RF-mutation into one heavy chain of a bispecific (Figure 2C) [34]. This results in three species after production, two homodimers and one heterodimer that can be separated by Protein-A chromatography. From a statistical point of view, 25% of the transfection products will carry the double mutation in both heavy chains and will not interact with the Protein-A column during purification. Consequently, this unwanted side product can be found in the flow-through. Another species with approximately 25% of the total expression product carries no mutations in their heavy chains and thus, will strongly bind to Protein-A. The main species being about 50% is the desired bispecific comprising the RF double mutation in just one chain, which displays medium affinity to the resin. By application of a pH gradient, the medium and strong binders can be separated. However, a specific Protein-A based resin needs to be used (MabSelect SuRe, GE Healthcare), which lacks V_H_ binding to the column. In addition, it needs to be mentioned that from a rough estimate, 50% of the product is composed of undesired protein, which may impact the overall developability of such molecules [34].

Recently, a novel approach for generating bsAbs based on common light chains and the stable IgG1 architecture was published by de Kruif and colleagues [35]. For this, double mutations are incorporated into two antibody heavy chains. The “DEKK” pairs consist of substitutions L351D and L368E in one chain, whereas the other chain harbors L351K and T366K exchanges. The formation of salt bridges between lysine residues as well as native and substituted amino acid residues on the opposite CH3 stabilize the DEKK heterodimer (Figure 2D). MCLA-128, which is a bispecific DEKK antibody targeting Her2 and Her3, is already evaluated in several clinical trials for the treatment of breast, gastric, non-small cell lung and other cancers.

Novimmune’s kappa-lambda (κλ) body platform is different to all other methods [36]. The allurement of this method lies in the fact that the heavy chains remain unchanged. To introduce bispecificity, a fixed heavy chain is combined with a lambda or kappa light chain naïve or synthetic antibody repertoire, which was followed by selections for target binding via phage display. Herein, the lambda library is panned against “antigen-A”, while the kappa library is panned against “antigen-B”. Subsequently, the common heavy chain is produced in HEK293 or CHO cells together with both the lambda and kappa light chain. A three-step purification process via Protein-A, Kappa- and Lambda-select columns allows the isolation of pure kappa–lambda bodies (Figure 2E). Similar to the RF-mutation, about 50% of produced antibodies are mispaired side products (i.e., kappa-kappa and lambda-lambda antibodies) [36].

In addition to the described technologies, a variety of other strategies can be found in literature, such as electrostatic steering approaches [37], Fab-arm exchange [38,39,40] and cleavable leucine zipper [41], which are comprehensively described elsewhere [2].

## 4. Methods for Specific Light Chain Association

The most obvious method to assure cognate heavy and light chain pairing is the use of a “common light chain”, which is essentially the identical light chain in both Fab-arms of a bispecific [2]. In 1995, Sally Ward generated common light chain monospecific antibodies by combining heavy chain repertoires after a mouse immunization (with Vα1934.4) with the light chain of a lysozyme binding antibody in phage display libraries [42].

**Figure 3:**
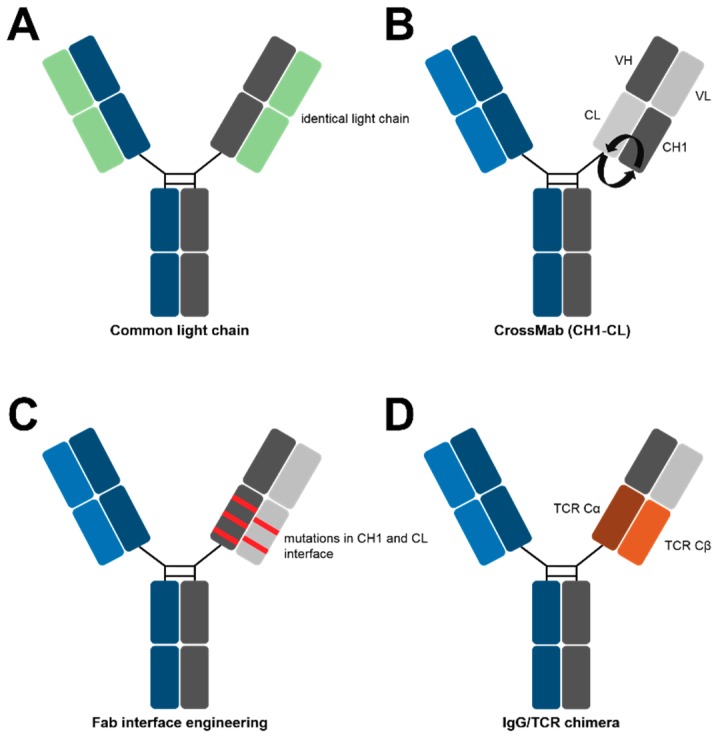
Methods for cognate light chain pairing in IgG-like bsAbs.

In the context of bsAbs, Genentech produced common light chain antibodies using phage display libraries with restricted light chain diversities [30]. After selection against different targets, antibodies with identical light chains were chosen and manufactured with KIH heavy chains (Figure 3A). Moreover, our group published a strategy to identify common light chain binders from immunized transgenic rats, harboring fully human variable antibody regions [43]. Yeast Surface Display (YSD) libraries were generated from the heavy chain repertoire of immunized rats and were combined with a randomly chosen distinct light chain or the light chain of a therapeutic antibody. Selections against different targets yielded several common light chain bispecifics.

Apart from common light chain approaches, other strategies integrate two existing antibodies into the bispecific format. One of the most prominent examples is Roche’s Crossmab technique, which was initially described in 2011 [44]. Here, domain swapping leads to a crossover between heavy and light chains on one side of the bsAb. As a result, only the corresponding light chain with accordingly swapped domains can bind to the swapped heavy chain, while the unchanged native light chain cannot. Originally, three different variants were tested: one that swaps the entire Fab; one that swaps V_H_ and V_L_; and one that exchanges CH1 and CL. While several side products were formed in the Crossmab-Fab and Crossmab-V_H_-V_L_, no significant mispairings were observed with the CH1-CL Crossmab (Figure 3B). As of today, four of these entities have entered clinical trials [44].

In a very recent study, it was shown that the introduction of a minimal set of amino acid mutations into the CH1-CL interface leads to the predominant formation of cognately paired heavy and light chains (Figure 3C) [45]. To this end, a rational design strategy was utilized to adapt repulsing CH3 residues in heterodimerized heavy chains to the respective CH1-CL interface. The alignment of CH3 and CL revealed that several previously mutated CH3 positions were homologous in the CL. Extensive rational design subsequently led to the prediction of four novel CH1-CL interfaces (with different mutations), which all promote the correct assembly of different light chains to their respective heavy chain. Some of the designs increased cognate pairings up to 99%. However, other groups demonstrated the ability of interface engineering to drive specific heavy light chain pairing [46,47]. In this study, a maximum of only two residues have been mutated per chain, which did not negatively affect the structure and function of the bsAbs [45].

Another interesting approach for cognate heavy and light chain pairing was described by Demarest and colleagues [48]. Here, in one Fab arm, the constant domains CH1 and CL were substituted by constant T-cell receptor domains Cα and Cβ, forcing partial heavy and light chain assembly (Figure 3D). However, additional linker optimization between the variable and chimeric constant domains as well as a loop replacement was needed to obtain a quantitative pairing [48].

Other technologies are based on an engineered disulfide bond in one CH1-CL interface [49], which includes computational and rational interface engineering [50,51].

Additionally, a plethora of different strategies has been described that differ significantly from the classical antibody domain structure and assembly, such as rat/mouse quadromas, use of scFvs and single chain Fab fragments (scFabs). A comprehensive review also describing non-IgG-like bispecifics was recently published by Brinkmann and Kontermann [52].

## 5. Conclusions

During the last two decades, intensive progress has been made in the field of bsAb engineering. Nowadays, a plethora of different technologies is available to construct bsAbs in a IgG-like format. Emicizumab, which is a common light chain IgG-like bispecific antibody, has been approved recently as a therapeutic agent and many antibodies are currently under preclinical and clinical investigations. Well-established technologies for heterodimeric heavy chain pairing, such as the knob-into-holes strategy, can be used freely today after patent expiry. This might pave the way for new developments in the bispecific antibody field, which can also be driven by smaller biotech companies. However, in many modes of action, bispecific molecules still need to demonstrate their superiority, which is their clinical benefit in comparison to antibody mixtures or monospecific antibodies. It will be interesting to see in the future whether the promises made by “next generation therapeutics”, such as bsAbs, will translate into a more effective clinical outcome for patients in various indications.
